# Atlantic water intrusion triggers rapid retreat and regime change at previously stable Greenland glacier

**DOI:** 10.1038/s41467-023-37764-7

**Published:** 2023-04-19

**Authors:** T. R. Chudley, I. M. Howat, M. D. King, A. Negrete

**Affiliations:** 1 Byrd Polar and Climate Research Center, Ohio State University, Columbus, OH USA; 2 School of Earth Sciences, Ohio State University, Columbus, OH USA; 3 Polar Science Center, University of Washington, Seattle, WA USA; 4 Present Address: Department of Geography, Durham University, Durham, UK

**Keywords:** Cryospheric science, Climate change, Physical oceanography

## Abstract

Ice discharge from Greenland’s marine-terminating glaciers contributes to half of all mass loss from the ice sheet, with numerous mechanisms proposed to explain their retreat. Here, we examine K.I.V Steenstrups Nordre Bræ (‘Steenstrup’) in Southeast Greenland, which, between 2018 and 2021, retreated ~7 km, thinned ~20%, doubled in discharge, and accelerated ~300%. This rate of change is unprecedented amongst Greenland’s glaciers and now places Steenstrup in the top 10% of glaciers by contribution to ice-sheet-wide discharge. In contrast to expected behaviour from a shallow, grounded tidewater glacier, Steenstrup was insensitive to high surface temperatures that destabilised many regional glaciers in 2016, appearing instead to respond to a >2 °C anomaly in deeper Atlantic water (AW) in 2018. By 2021, a rigid proglacial mélange had developed alongside notable seasonal variability. Steenstrup’s behaviour highlights that even long-term stable glaciers with high sills are vulnerable to sudden and rapid retreat from warm AW intrusion.

## Abstract (web summary)

From 2018 to 2021, KIV Steenstrups Nordre Bræ, a marine-terminating outlet glacier of the Greenland Ice Sheet, retreated ~7 km, thinned ~20%, doubled in discharge, and accelerated ~300%. This rate of change is unprecedented in the observational record.

## Introduction

The Greenland Ice Sheet is the dominant contributor to global sea-level rise from the cryosphere, losing 222 ± 30 billion tonnes of ice per year between 2012 and 2017^1^. Between a half and two-thirds of loss since the 1990s has been attributed to acceleration in ice discharge from marine-terminating outlet glaciers^1–3^, a process initiated through interactions between the ocean and the glacier terminus^4–6^. Understanding these interactions is a critical component of understanding future sea level contributions from the Greenland Ice Sheet^6^.

Forcing at the ice–ocean interface is understood to occur via three primary mechanisms. The first is the submarine melting of glacier termini by the transport of warm, deep Atlantic water (AW) through fjords to glacier calving fronts^5,7,8^, initiating calving loss by submarine melt and undercutting of the ice front^9–12^. The second is terminus melt initiated by near-ice circulation and plumes from fresh subglacial discharge^13,14^, which enable heat transfer between the ocean and ice. Discharge is sourced from the surface melt of the ice sheet, leading to both oceanic and atmospheric influences on terminus melting^15^. The third mechanism is the acceleration of the calving rate via the breakup rigid ice mélange^16–21^, the backstress of which acts to inhibit calving^22–24^. All three mechanisms have the potential to trigger rapid retreat in previously stable glaciers, sustained by positive feedback arising from retrograde bedslopes^25^ and dynamic thinning^26,27^.

However, glaciers exhibit highly heterogeneous responses to relatively uniform ocean forcing^2,3^, even when directly adjacent^28–30^. This variability has been attributed to fjord geometry^12,31^, glacier geometry^23,26,32,33^, subglacial hydrology^34^ and the distribution of oceanographic currents^7,35^. None of these, however, can consistently explain spatiotemporal heterogeneity in glacier response, suggesting that such variability is a complex combination of multiple conditions. For instance, some have argued that deep termini, susceptible to buoyant flexure and losing mass through full-thickness calving, are controlled by seasonal^18^ and interannual^19^ mélange variability, whilst retreat of shallow glaciers, calving primarily through small-magnitude serac failure, is driven primarily by subglacial melt^6,21^. Conversely, others have suggested that the deep termini are forced by AW intrusion, whilst shallow, well-grounded glaciers are protected by their proglacial bathymetry^12,31,36^. The former has been supported by glacier-scale studies of deep outlets such as Sermeq Kujalleq (Jakobshavn Isbræ) and Kangerlussuaq^19,20,37^, which conclude that retreat was initiated by the destabilisation of rigid winter mélange. Meanwhile, other studies have found deep outlets, such as Zachariae Isstrøm^29^ and Helheim^5^, to be forced primarily by AW. Sermeq Kujalleq (Store Glacier) has been found to be susceptible to both processes^38^, destabilising entirely in response to either a doubling of frontal melt or a complete loss of mélange. This is problematic for larger-scale modelling exercises, which frequently choose only one mode of ice–ocean interaction to parameterise^2,8^. Being able to better differentiate the controls on tidewater glacier vulnerability is important as many glaciers that contribute significantly to Greenland’s cumulative ice discharge are less well studied than the few that dominate the literature^2^.

Diverse forcing has typified Greenland’s southeast sector. A notable increase in ice discharge beginning in ~2001 that extended as far as 69°N was attributed to AW^5,7,12^, corresponding with the latitudinal extent of the warm subtropical waters carried by the Irminger Current^7,35^. Retreating glaciers were typified by deep fjords (allowing AW access) and retrograde bedslopes^31^. However, a more recent synchronised retreat in response to atmospheric warming began across the sector in 2016^39^, including at Kangerlussuaq^19,40^. Studies of ocean reanalysis data concluded that this response was not due to AW, which experienced no anomaly in 2016^19,39^. Instead, it was proposed that the retreats occurred in response to either (1) atmospheric forcing leading to a high cumulative meltwater input, resulting in the increased submarine melt at the front^39^; or (2) surface-level ocean forcing resulting in a loss of winter rigid mélange, leading to increased calving^19^. Once again, understanding controls on tidewater glacier vulnerability is necessary for both identifying the climate and ocean conditions that lead to past retreats and for predicting future change.

**Fig. 1:**
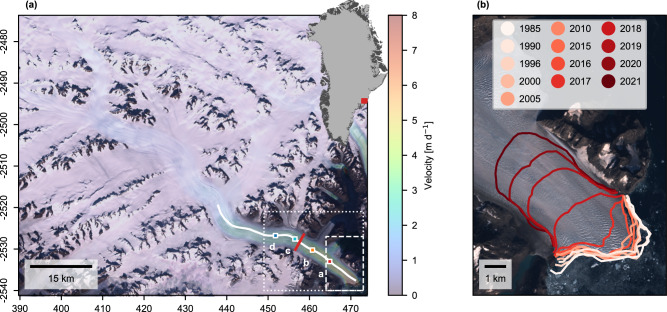
Location of Steenstrup. **a** Location and speed of KIV Steenstrup Nordre Bræ. The colour scale indicates the mean 2016 velocity from ITS_LIVE velocity pairs. Coloured squares a–d indicate locations used to sample velocity time series in Fig. 2, the white line marks the centreline used to derive profiles in Fig. 3, and the red line marks the flux gate used for ice discharge calculation. The dotted box marks the extent of Fig. 4, and the dashed box marks the extent of panel (**b**). The background is a composite of median Sentinel-2 RGB pixel values from May to October 2016. Coordinates in unit kilometres of NSIDC Polar Stereographic North. Inset shows the location of Steenstrup within Greenland. **b** Changing front position of Steenstrup since 2016, identified using GEEDiT^82^.

Here, we examine recent changes at K.I.V Steenstrups Nordre Bræ (66.53°N, 34.57°W; Fig. 1a; hereafter Steenstrup), an outlet glacier of the southeast Greenland Ice Sheet that exhibited long-term stability until a large destabilisation in 2018. We use observations and reanalysis products to outline the extent of change and understand the underlying mechanisms, identifying the sensitivities of the glacier to forcing out and outlining how this sensitivity changes through time.

## Results

### Temporal changes at Steenstrup

**Fig. 2:**
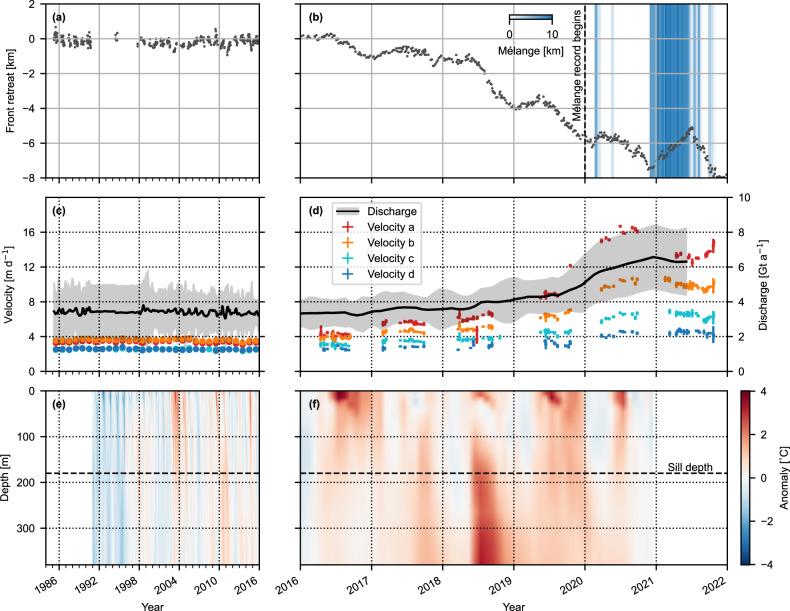
Time series of changes at Steenstrup. Front position between **a** 1985 and 2015 and **b** 2016 and 2022, with blue shading denoting the along-fjord extent of the rigid mélange measured from the glacier terminus between 2020 and 2021 (a zoomed 2020–2021 version of the panel (**b**) is shown as Supplementary Fig. S1). Black dashed line marks beginning of the mélange record (January 2020). Ice discharge (black curve with shading as 2σ uncertainty) and annual velocity (coloured points with error as median reported ITS_LIVE error of sample zone) between **c** 1985 and 2015 and **d** 2016 and 2022. Point colours refer to points in Fig. 1a. Mean ocean temperature anomaly from CMEMS Arctic Ocean Physics Reanalysis monthly mean data for the CE1 sample zone (Supplementary Fig. S5) between **e** 1992 and 2015 and **f** 2016 and 2021. The horizontal dashed black line refers to the absolute lower limit of Steenstrup’s proglacial sill. An expanded version of the 2020–2021 mélange data is included as Supplementary Fig. S1, and an expanded version of panels (**a**), (**c**), and (**e**) as Supplementary Fig. S2.

Prior to 2018, Steenstrup’s calving front had been stable for decades, with an average 2015 front position only ~200 m from the average 1985 front position (Fig. 2a). The average speed at the calving front was ~7 m d^−1^ (~2.5 km a^−1^) (Fig. 1a), with an ice discharge of 3.34 Gt a^−1^ in 2016, putting it in the 82nd percentile of glaciers by contribution to Greenland’s ice discharge^3^. Seasonal variability in the front position was low, with a standard deviation of 155 m. In most years, the front exhibited a negligible seasonal advance or retreat, with only a few exceptions to this rule (2002, 2009, 2010, 2014, 2016), where ~0.8–1.0 km of retreat occurred. This temporary retreat generally began in June but recovered, often overwinter but always within ~2 years. For instance, between early June 2016 and the 16th of January 2017, Steenstrup’s calving front retreated by ~1.3 km before recovering ~0.7 km between January and June 2017.

In contrast, 2018 saw more significant and sustained retreats. This began in mid-May, totalling ~3.2 km by the 12th of January when the annual winter advance began. This pattern repeated in 2019, retreating ~3 km between mid-May and early February. After a relatively short advance period relative to 2018, an additional ~2.1 km of retreat occurred between early May and November 2020. At this point, the front began advancing, far earlier than in previous years and lasting much longer into July 2021. By 4th July 2021, the terminus had advanced to the annual maxima, ~2.4 km from the November minima, and only ~0.3 km beyond the 2020 maxima. This advance was matched by a significant late-summer retreat of 2.9 km by early December, ultimately losing ~0.5 km relative to the annual minima of the previous year. In total, Steenstrup retreated ~7.1 km between 2018 and 2021, resulting in the creation of a new fjord ~6 km long.

Steenstrup’s retreat occurred alongside an associated increase in glacier speed. After exhibiting no change between 1985 and 2016 (Fig. 2b, c), speed increased at all sample points between 2016 and 2020. The frontmost sampling point reached a maximum of 16.8 m d^−1^ in August 2020 (an increase of more than 270%). However, alongside the front advance between November 2020 and July 2021, Steenstrup’s speed greatly reduced, declining to ~12.8 m d^−1^ at the frontmost point by early July. At the point closest to the glacier front, acceleration occurred coincident with the retreat of the glacier calving front and continued for the rest of the year, reaching a maximum of 15.0 m d^−1^ in the last observation of the year (2021-10-22). However, it took time for this acceleration to propagate inland, with the two points further inland not accelerating until late August/early September, whilst the point furthest from the terminus showed no clear late-season acceleration. The total increased flow speed resulted in a doubling in the rate of ice discharge by 2021, reaching 6.37 Gt a^−1^ and placing Steenstrup in the 93rd percentile of Greenland’s outlet glaciers by contribution to total ice discharge, up from 3.34 Gt a^−1^ and 82nd percentile in 2016^3^.

**Fig. 3:**
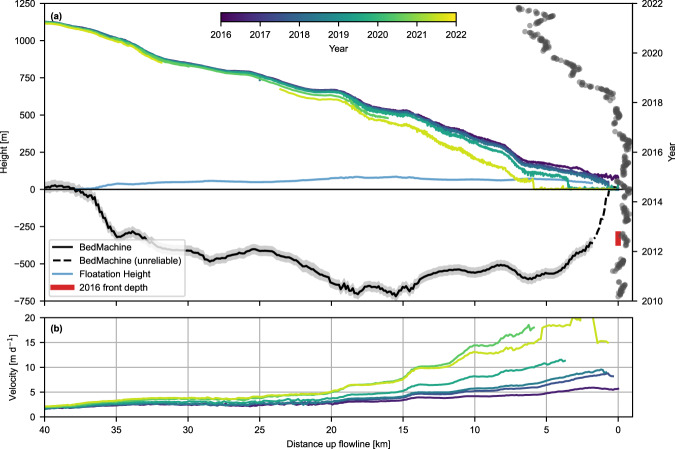
Profile of changes at Steenstrup. **a** Surface (ArcticDEM strips) and bed elevation (BedMachine v4) along the profile shown in Fig. 1a. Blue line shows the floatation height of the ice column. Grey dots mark terminus positions through time. The red bar marks the vertical range of the 2016 terminus depth from Oceans Melting Greenland Multi Beam Echo Sounder data^43^. Note that the front 1.8 km of the BedMachine data, denoted with a dashed line, is determined to be unreliable based on OMG and Operation IceBridge data (see Supplementary Text). **b** Annual speed profiles from ITS_LIVE velocity data 2016–2021 along the profile shown in Fig. 1a.

**Fig. 4:**
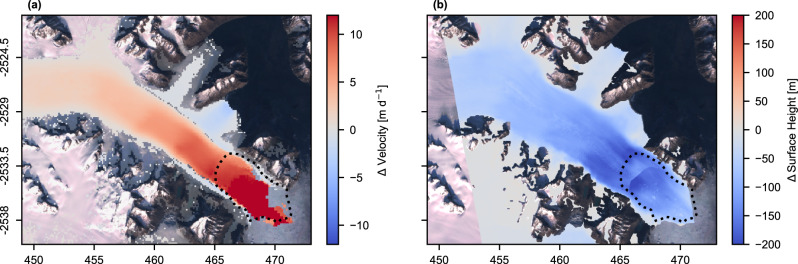
Spatial extent of changes. **a** Difference between the weighted mean average of 2021 and 2016 ITS_LIVE velocity pairs. Colours are translucent where change is not significant. **b** DEM difference between ArcticDEM strips captured on 2016-03-27 and 2021-07-31. Dotted lines mark terminus positions on 2016-09-28 and 2021-10-29. Coordinates in unit kilometres of NSIDC Polar Stereographic North.

Sampling annual velocity mosaics along the flowline (Fig. 3b) provides further information about the spatial extent of speed increases. Statistically significant increases in speed between 2016 and 2021 are visible up to 40 km inland, where speed increased 24% from 1.7 m d^−1^ in 2016 to 2.1 m d^−1^ in 2021. On average, speed increased over 100% within 19 km of the 2016 front position, over 50% within 27 km, and over 20% within 40 km. This acceleration was limited to the main glacier trunk: both distributaries exhibited statistically significant slowdowns, the northernmost distributary from ~0.5 m d^−1^ to ~0.2 m d^−1^, and the southernmost from ~4.4 m d^−1^ to ~0.9 m d^1^ (Fig. 4a). This contrasting behaviour between the main outlet and distributaries was associated with a significant shift in the medial moraine of the northernmost distributary as the flow was captured by the main outlet (Supplementary Fig. S3).

Steenstrup’s retreat was also associated with a significant reduction in surface elevation. Between 2016 and 2018, the surface of Steenstrup lowered by ~10–20 m a^−1^ within ~6 km of the 2016 front, with the rate of lowering decreasing to 1–2 m a^−1^ by 20 km inland (Fig. 3a). However, after 2018, surface lowering accelerated and propagated inland rapidly. Between 8 and 10 km upglacier of the 2016 terminus, losses approached 50 m a^−1^ between 2018 and 2021. The Regional Atmospheric Climate Model (RACMO2.3p2) surface melt product^41^ models the maximum annual melt at Steenstrup for the 2018–2021 period between 5.0 and 7.6 m a^−1^, suggesting surface elevation change is far greater than could be attributed to increased surface melting and hence likely related to ice thinning under increased along-flow strain rates (i.e., dynamic thinning). By 2021, a combination of surface elevation loss and retreat along a retrograde bedslope resulted in ~1 km of the tongue being at or near flotation by 2021, as indicated by the ice surface height relative to flotation (Fig. 3a). Total elevation losses exceeded 200 m between 2016 and 2020 (Fig. 4b). Surface losses also occurred in the distributaries between 2016 and 2021: up to 40 m at the terminus of the northern distributary and 60 m at the southern distributary.

### Forcing

**Fig. 5:**
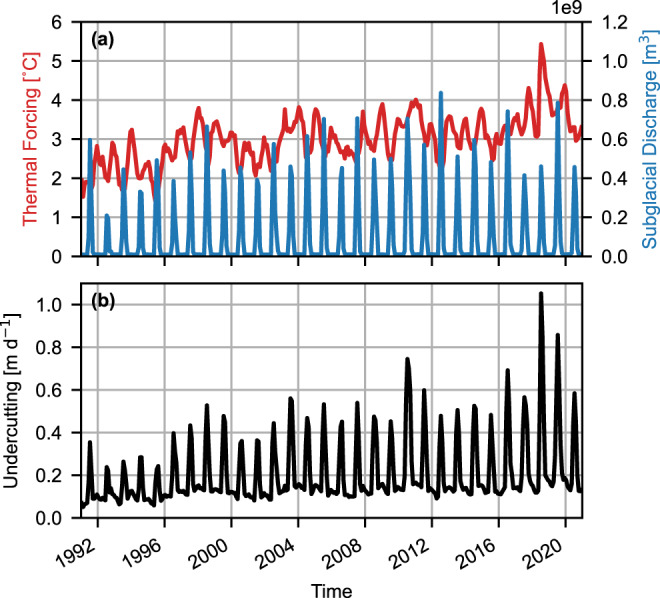
Undercutting melt rate modelling results. **a** Time-varying inputs to thermal forcing parameterisation: the depth-averaged ocean thermal forcing in the lower 60% of the water column (red line) and the integrated monthly subglacial discharge of Steenstrup’s hydrologic basin (blue line). **b** Modelled monthly average undercutting melt rate across the submerged calving face in m d^−1^.

Despite the generally stable front position, the post-2000 period displays several seasonal examples of retreats in front position on the scale of hundreds of metres (e.g., 2003, 2009, 2010, 2014, and 2016). Full recovery took one or two winter seasons. These ephemeral retreats occurred alongside modelled periods of positive surface temperature anomalies in climate reanalysis datasets. This is true in oceanic and atmospheric products, with warm surface waters in the proximal continental shelf zone from the Copernicus Marine Environment Monitoring Service (CMEMS) Arctic Ocean Physics Reanalysis monthly mean data^42^ (Fig. 2e, f), and high-modelled ice surface melt across the Steenstrup discharge basin in the RACMO2.3p2 surface melt product (Fig. 5a).

Retreat initiated in 2018 was synchronous with an exceptionally warm and thick temperature anomaly in the CMEMS ocean reanalysis model that reached within 100 m below the sea surface (Fig. 2f). At the deepest reanalysis level (380 m), this anomaly reached a maximum of +3.3 °C in July 2018 (the second-highest annual maxima, in October 2017, was only +1.3 °C). At 186 m—the reanalysis depth above which water could overtop the proglacial sill according to Oceans Melting Greenland Multi Beam Echo Sounder (OMG MBES) bathymetry data^43^ (Supplementary Fig. S4a)—the July 2018 anomaly was +2.4 °C. In contrast, the 2018 event did not coincide with exceptional positive anomalies in modelled near-surface water temperature (Fig. 2f) or subglacial discharge driven by modelled surface melt (Fig. 5a).

Modelled undercutting melt rate peaked at an all-time high in July 2018 at 1.05 m d^−1^ (Fig. 5b), with the second-highest peak levels following in July 2019 at 0.86 m d^−1^. Supporting our above inferences, the 2018 record was driven by the record high in ocean thermal forcing rather than rates of subglacial discharge, which in 2018 was at only 86% of the long-term average annual high (Fig. 5a). Historic anomalous undercutting rates in 2003, 2010, and 2016 align with temporary retreats in terminus front position, and appear to be driven by a combination of thermal forcing (e.g., 2003) and subglacial discharge (e.g., 2016).

## Discussion

Both this study and others indicate that Steenstrup has been stable on a decadal time scale prior to 2018, with very little seasonal variation going back to at least the 1980s (Fig. 2a, b). The front position further remains broadly the same in 1966 declassified satellite imagery^44^ and 1938–1942 cartographic records^45^, and a push moraine attributed to a 20th-century regime^46^ is present only 200 m from the 2016 front position (Supplementary Fig. S4a). Advance beyond this point was likely negated by an increase in exposed calving face to the ocean, whilst any retreat was stabilised by the front position on a ridge^31,33^.

The 2018–2021 destabilisation was considerable in magnitude and extent. There was no significant precursor activity, suggesting a sudden external forcing. Steenstrup retreated ~7 km, thinned at nearly 50 m a^−1^, quadrupled in speed, and doubled in ice discharge. Whilst tidewater glacier retreat rates greater than 1000 m a^−1^ have precedent^47^, this retreat rate is still among the largest observed, with most glaciers retreating <200 m a^−1 ^^48^. The dynamic thinning rate exceeds that of Kangerlussuaq^40,49^ and matches that of Sermeq Kujalleq during the early 2000s^4,50,51^. A quadrupling in speed within 5 years is, to our knowledge, unprecedented among the relative accelerations of large Greenland glaciers^52^, including the doubling of velocity over 5 years of Sermeq Kujalleq in the early 2000s^50^. The short-term doubling of ice discharge is likewise unprecedented and only exceeded by Harald Moltke Bræ, which ~tripled its annual ice discharge within the span of a decade^3^. However, this is explained by the glacier entering a surge phase rather than as a response to external forcing^53^. Hence, recent events at Steenstrup are proportionally comparable to, or exceed, the largest instances of tidewater glacier change across the Greenland Ice Sheet. It has been suggested^52^ that rapid and large destabilisation may be limited to only a few glaciers. Steenstrup indicates that other, apparently stable, glaciers may still be primed to retreat under ever more positive forcing^54^.

Previous studies have attributed the post-2016 retreat of Greenland’s south-eastern tidewater glaciers to two mechanisms. The first mechanism identified warm surface temperatures in 2016 as a primary forcing across Southeast Greenland^39^, with summers of high cumulative meltwater driving terminus melt via subglacial discharge^15,21^. However, although high surface melt and subglacial discharge occurred at Steenstrup in 2016 (Fig. 5a), the terminus response was comparable to other years with above-average surface air temperatures (e.g., 2002, 2009, 2010, and 2014; Fig. 2a, b; Supplementary Fig. S2). Steenstrup’s 2017 advance indicates a trajectory to recover its front position in line with previous seasonal retreats. Hence, Steenstrup is not obviously sensitive to high surface air temperatures, even though shallow, well-grounded glaciers have been hypothesised to be particularly vulnerable to such events^21^. We suggest that Steenstrup’s geometry, with a confined and narrow trunk ~100 km long, reduces the catchment area for surface melt runoff and increases subglacial transport times to the terminus. This limits the rate and magnitude of subglacial meltwater discharge and hence induces terminus melt.

The second mechanism is attributable to the loss of rigid mélange in response to warm surface waters, leading to a loss of backstress or impediments to calving and a subsequent increase in ice discharge. This mechanism was observed at Kangerlussuaq, with a significant retreat following a lack of mélange development in the winters of 2016/17 and 2017/18^19^, a hypothesis later supported by numerical modelling^37^. However, there is a historic lack of mélange at Steenstrup. The optical satellite and ITS_LIVE velocity record show that, prior to 2018, a small amount of mélange (on the order of hundreds of metres) was observed in a small embayment on the southwest side of the terminus only, negating the ability of the mélange to modulate the timing and magnitude of calving, and relatedly, ice discharge.

Without enhanced surface melt and mélange loss as causal factors of Steenstrup’s retreat, terminus melt from warm AW intrusion remains the most common alternative hypothesis^5,7,8,29^. OMG MBES observations (Supplementary Fig. S4a) indicate a lower limit of the LIA proglacial sill at ~180 m. AW along the East Greenland Shelf primarily occurs beneath depths of ~250 m^55^, above which cold and fresh polar water dominates. AW presence across the shallow coastal shelf in front of Steenstrup (maximum depths of ~400 m) is rare compared to the front of Sermerlik and Kangerlussuaq Fjords, which have deep canyons to aid AW transfer^55^ (Supplementary Fig. S5). This is evident when comparing modelled temperature anomaly data between the sites (Supplementary Figs. S5 and S6). However, warm modes exist whereby AW is incorporated into the water column at Kangerlussuaq Fjord, where deep canyons aid AW transfer^55^ (Supplementary Fig. S5), and is carried south across the coastal shelf as part of the East Greenland Coastal Current^56–58^. Indeed, the CMEMS reanalysis product suggests that an unprecedented warm anomaly occurred within the CE1 sample zone up to ~100 m depth in 2018, coincident with the unstable retreat of Steenstrup and well in excess of the ~180 m sill depth (dashed line in Fig. 2e, f). Given the limitations of reanalysis modelling, it is desirable to validate this with in situ data. OMG CTD data are limited in the vicinity, and the available CTDs indicate a high degree of spatial heterogeneity (Supplementary Fig. S9). However, we also find that CTD site 144, located only ~40 km from the Steenstrup terminus, exhibits warm (>4 °C) water extending up to a depth of 130 m in August 2018. This is the shallowest water column depth that warm water reached during the 2017–2020 site 144 CTD record (although CTD timings were inconsistent between years, ranging from August–October), suggesting AW intrusion was high proximal to Steenstrup in 2018. An anomalous 2018 AW intrusion was previously reported at Sermilik Fjord^59^, who inferred that AW extended along the entire trough year-round in 2018. Our conclusions are limited by the lack of observational thermal profiles from within the fjord that would provide in situ validation of AW intrusion, but modelling results and data presented are supportive of a hypothesis where, in 2018, AW could access even the shallowest sections of the coastal shelf in front of Steenstrup, overtopping the sill and inducing retreat.

If ocean reanalysis data is representative, our melt parameterisation indicates that ocean forcing alone is sufficient to induce all-time highs in undercutting melt rates of 1.05 m d^−1^ in 2018 (Fig. 5a cf. 5b). This magnitude is consistent with the simulated melt rates of 1–2 m d^−1^ at Umiammakku Isbrae and Kangilerngata Sermia alongside their retreats in the early 2000s^10^. In contrast, in the high surface melt year of 2016, peak undercutting was 34% lower (0.69 m d^−1^). The vulnerability of Steenstrup, which was relatively shallow and well-grounded at the ice front, to AW rather than mélange destabilisation or surface melt contrasts with expectations that AW is least effective at initiating retreat in shallow, well-grounded glaciers^12,21^. Indeed, Steenstrup is identified as a stable ‘calving ridge’ glacier^12^, raised above the level of AW by a sill and having only a small floating section. It is of note, however, that this interpretation may be skewed by the poor topographic reconstruction of the terminus region (see Supplementary Text). Increasing warming of waters around Greenland since the mid-1990s^5^ may result in increasingly common AW access to marine-terminating glaciers thought to be protected by shallow shelves and sills. However, predicting which are vulnerable may be challenging due to the poor knowledge of bed topography and proglacial bathymetry at many of the lesser-studied glaciers in Greenland.

Following destabilisation, Steenstrup retreated rapidly (~2 km a^−1^) between 2018 and 2020 down a retrograde bedslope (Fig. 3a) until the front stabilised at a second sill in 2021. Following this retreat, Steenstrup began displaying a high seasonal variability in terminus position, advancing significantly in the 2020/21 winter before retreating in summer 2021. This advance/retreat pattern approximates the ‘type b’ glacier terminus behaviour^21^, attributed to the seasonal formation and breakup of mélange. This interpretation is supported by the mélange extent data (Fig. 2b), which indicates that mélange was extensive in the 2020/21 winter but absent in 2019/20, and further supported by annual velocity mosaics, which record fast-flowing (>16 m d^−1^) mélange in the 2021 mosaic but not 1985–2020 (years 2016–2021 visualised in Fig. 3b). By the 2020/21 winter, the retreat of the terminus ~6 km from fjord edge enabled mélange to form fast to the fjord margins, inducing backstress on the glacier front and suppressing calving rate^60^. This was aided by the glacier thinning sufficiently that the terminus 1 km from the front was at or near floatation^61^ (Fig. 3a), as well the fact that mélange buttressing has been shown to increase with the length-to-width ratio of the fjord^22,24^. We hypothesise that these changes meant that, by 2021, the balance of stresses acting on the glacier terminus were more sensitive to the increased mélange backstress in the absence of reduced basal traction, modulating the new emergent seasonal behaviour.

Steenstrup’s retreat halted in 2021, likely due to a combination of reaching a prograde bedslope (Fig. 3a), a reduction in driving stress due to rapid dynamic thinning, and the development of a rigid proglacial mélange. However, with velocities quadrupled since 2016, Steenstrup likely remains out of balance. Ice flow is confined to relatively narrow valleys and catchments until connecting to the ice sheet over 100 km upstream, limiting influx (Fig. 1a). As such, diffusive acceleration is concentrated in the trunk, increasing rates of dynamic thinning relative to less confined glaciers^52^. In this sense, the upstream response to retreat is similar to Alaskan tidewater glaciers, such as Columbia Glacier^62^. Rapid thinning is resolvable tens of kilometres inland (Fig. 3a), and this imbalance in ice discharge will likely persist due to a transition to a deeper terminus that is at or near floatation (Fig. 3a). This has been suggested to enhance tabular calving driven by full-thickness fracture^21,63^ rather than smaller, sub-aerial calving events, a transition reported at Columbia^62^ and Bowdoin^64^ Glaciers. We hypothesise that these factors make the 2021 terminus position untenable in the medium term, even accounting for the current position on a bedrock bump (Fig. 3a).

The next ~8 km of the retreat will be influenced by the collapse of the two tidewater distributaries, which are already thinning rapidly (Fig. 4b). As these areas are also decelerating (Fig. 4a), we suggest that this is not due to dynamic thinning but instead due to decreasing influx resulting from flow capture by the main trunk, as indicated by changes to the medial moraine (Supplementary Fig. S3). Once the main terminus retreats past the distributaries, they will rapidly disintegrate due to submarine melt and calving. However, their collapse will once again restrict the ability of a rigid mélange to form by reducing the length-to-width ratio of the fjord area available^22,24^ due to a lack of fjord margins on the northeast side. This will enhance the calving rates of the main trunk and further enable retreat. The basal topography record is poor in this sector (see Supplementary Text), but visual analysis of airborne radar data (Supplementary Fig. S7) suggests that retrograde bedslopes likely continue to occur, with some rises that may or may not stall retreat^65^, until approximately 20 km from the 2016 calving front. The extent to which the glacier stabilises between this point and when the bed reaches sea level (35 km inland) will be controlled by the full extent of dynamic thinning and ocean forcing.

Retrograde bedslopes, imminent destabilisation, loss of distributaries, and continued dynamic thinning all indicate further rapid retreat of Steenstrup. However, the relative influence of atmospheric and oceanic forcing may modify the rate of response, especially at prograde bedrock rises. As identified above, Steenstrup’s seasonal behaviour in 2021 suggests a new sensitivity to mélange variability and, thus, near-surface temperatures^19,37^. Meanwhile, sensitivity to warm AW intrusions will continue, especially if the collapse of the two northeastern distributaries provides new pathways for AW entry through the deeper northern fjord (Supplementary Fig. S4b). The future sensitivity of the glacier to surface melt and resultant subglacial discharge is less clear. However, a quadrupling in velocity may result in an increase in subglacial melt generated via frictional heating at the bed, whilst the increased positive surface strain rates in accelerating zones may provide new pathways for water to reach the bed through crevasses. As a result, the changes occurring at Steenstrup may make it more vulnerable to the full range of tidewater glacier retreat mechanisms.

Steenstrup provides a unique example of rapid glacier change that has occurred entirely within a period of intense observational study from various Earth observation instruments. Occurring without resolvable precursor activity, the thinning and acceleration of Steenstrup propagating inland from 2018 were amplified by Steenstrup’s more confined geometry. This resulted in possibly the highest relative increase in glacier speed observed amongst fast-moving tidewater glaciers in Greenland, notably from a glacier previously thought to be stable and insensitive to external forcing. Our results indicate an unusual scenario where a shallow, grounded tidewater glacier was resistant to surface meltwater forcing but vulnerable to AW intrusion and highlight the potential development of sensitivity to warm surface temperatures (via mélange). Steenstrup should be a priority for ongoing in situ data collection, particularly the collection of in-fjord CTD data (to validate reanalysis-led hypotheses of warm water intrusion) and better basal topography (to better understand the future evolution of retreat). Aided by more comprehensive in situ data, Steenstrup is a good candidate for numerical modelling experiments: providing both the opportunity to test the ability of models to replicate complex changes and forcings and to provide future projections that will be falsifiable on a timescale of years.

Model reanalysis presented here and elsewhere^12^ that AW around the GrIS is becoming progressively warmer in recent decades^5^, providing increasing opportunities for warm waters to penetrate to the front of marine-terminating glaciers that are relatively more protected from incursions by proglacial bathymetry^54^. Steenstrup has shown that the stability of glaciers is hard to predict using our current knowledge of smaller, stable, and data-sparse glaciers, especially given the apparently poor reconstruction of the geometry of the ice–ocean interface at Steenstrup and elsewhere. There may be many more well-grounded and mélange-deficient glaciers that, whilst resistant to warm surface waters and subglacial discharge, are primed to retreat and increase their contribution to total GrIS ice discharge in the face of increasing AW incursion.

## Methods

### Calving front positions

Calving front positions were manually digitised along the centreline between 1985 and 2021 using Landsat 4–8, ASTER, and Sentinel-1 data, a continuation of the dataset previously presented by Walsh et al.^35^.

### Glacier velocity

We investigated the change in speed of Steenstrup with both scene-pair velocities (to construct time series) and annual velocity maps (to assess spatial change). To do this, we make use of ITS_LIVE velocity data^66,67^. Between 1985 and 2015, we downloaded annual mosaics of velocity provided by the ITS_LIVE project (Fig. 2c). Beyond 2016 (Fig. 2b), we downloaded all available scene-pair velocity data covering Steenstrup between 2016 and 2021 with >1% data coverage.

To present a time series (Fig. 2b), ITS_LIVE velocity pairs were sampled at locations between 6 and 23 km along the flowline from the 2016 calving front (Supplementary Table S1). We use 2016 as our pre-retreat standard across all observed variables as in 2017 the glacier was recovering from a minor retreat forced in late 2016 (see “Results” section: “Temporal changes at Steenstrup”), displayed a retreated terminus and slightly enhanced velocity relative to 1985–2016 norm (Fig. 2). We sample a 1 × 1 km box centred on the point, presenting the median speed and error of the sample zone where coverage is greater than 70%.

As updated annual mosaics between 2019 and 2021 were not available at the time of writing, we calculated our own annual mosaics to produce difference maps between 2016 and 2021 for visualisation (Figs. 3b, 4a). From the provided speed ($x$) and error ($\sigma$) values, we calculate the weighted mean ($\bar{x}$) as:

1 $$\bar{x}=\frac{\sum x/{\sigma }^{2}}{\sum 1/{\sigma }^{2}}$$

and the weighted standard error ($\bar{\sigma }$) as:

2 $$\bar{\sigma }=\frac{1}{\sum 1/{\sigma }^{2}}$$

A two-tailed unpaired *t*-test is used to assess whether differences between the mosaic pixels are significant (Fig. 4a).

### Mélange presence

Mélange velocities were derived for continuous 6-day periods throughout 2020 and 2021 from SAR imagery acquired in the interferometric wide swath (IW) mode from the Sentinel-1 A and B. Velocity maps over rigid mélange-occupied regions of the fjord and surrounding margin were processed following GrIMP workflows^68^, which measure 6-day displacements of features in image pairs by cross-correlating small (km-scale) image patches using a combination of speckle and feature tracking of the textured mélange surface. Here, we measure only the extent of rigid mélange, or regions of icebergs and sea ice that can be tracked from one image to the next because they maintain coherence, using the intersection of the rigid melange patch with an extended centre flowline (Supplementary Fig. S8). By contrast, the mapping algorithm fails if the mélange is non-rigid, or such that the individual constituents of the mélange move more randomly relative to each other. Based on this principle, we did not process prior to 2020, as no rigid mélange signatures were present in any ITS_LIVE mosaics between 1985 and 2020. When present, the extent of the rigid mélange is derived by comparing the distance between the outer limit of rigid mélange areas to the contemporaneous Steenstrup terminus position, which is manually traced at the same 6-day temporal resolution. Thus, in addition to understanding whether or not a rigid mélange is present adjacent to the terminus at a given time step, mélange extent provides a metric to evaluate potential relative back force at the calving front.

### Ice discharge

Monthly ice discharge from 1985 through 2021 was calculated through an upstream flux gate (Fig. 1), oriented perpendicular to ice flow across the width of Steenstrup. We sample ice thicknesses and ice velocities at 250-m-spaced coordinates along this gate. Velocities were obtained from both optical and SAR data, and surface velocity estimates are assumed to be representative of the depth-averaged velocity. These datasets included those derived from orthorectified optical imagery from LANDSAT 4, 5, 7, and 8, and ASTER bands 1–3 using MIMC2 algorithms^69^ and surface displacement mapping using SETSM^70^, as well as SAR products from Terra/TandemSAR-X. The velocity time series from King et al.^3^ were further appended through 2021 using velocities from GrIMP Sentinel 1 A & 1B^71^. Temporal data gaps are filled using a Kalman filter approach^72^ based on estimating a median seasonal variability computed from detrended available observations superimposed on the multiyear time series trend. Velocities are smoothed using a moving filter weighted by observational errors, which arise due to noise-to-pixel ratios and co-registration quality between paired images, and resampled at uniform monthly time steps. Ice thicknesses were obtained from differencing surface elevations from historic DEM data (ranging from AeroDEM through ArcticDEM) with bathymetry from BedMachine v4. Total ice thickness errors include the combined spatially variable and systematic errors from the BedMachine product with an estimated random error of 5 m from surface elevation data. Monthly ice discharge estimates were calculated by summing the product of ice thickness, velocity, and ice density (910 kg m^-3^) at each 250 m along-gate bin (Supplementary Fig. S8). Continuous discharge uncertainty bounds are derived from the standard deviation of a 1000-iteration Monte Carlo ensemble that perturbs the time series with random errors applied at each time step using errors bounds computed from combined velocity and thickness errors. We note that 2σ error estimates in the discharge values can be as high as 50% (Fig. 2c, d). This is largely reflective of greater uncertainties in velocities from the earlier LANDSAT record and from the high uncertainty in the bed profile across the flux gate, including limited radar flight lines and extrapolation across the glacier width, which is likely causing an unknown systematic under- or over-estimation in discharge. However, as change in discharge over the timescales of this study is overwhelmingly influenced by variation in ice velocity, any systematic variation due to poorly constrained bed topography will not impact on the timings or relative changes of discharge reporting.

### Topographic analysis

To assess elevation change over the period of interest, we use 2-m resolution ArcticDEM strips^73^. We manually identify high-quality strips between 2016 and 2012 (Supplementary Table S2), reference the heights to mean seal level using the EIGEN-C64 geoid provided as part of BedMachine v4^74^, and coregister the strips to the 2016 DEM^75^.

To explore the potential for floatation at the calving front, we estimate the theoretical floatation thickness (H_f) based on basal topography extracted from BedMachine v4 as

3 $${H}_{f}=-{h}_{b}\frac{{\rho }_{w}}{{\rho }_{i}}$$

where ${h}_{b}$ is the bed depth, and $\rho$_i_ and $\rho$_w_, are the densities of ice and water (assigned 917 and 1027 kg m^−^^3^), respectively.

### Ocean reanalysis data

We extract ocean potential temperatures from 1991 to 2020 from the Copernicus Marine Environment Monitoring Service (CMEMS) Arctic Ocean Physics Reanalysis monthly mean data^42^. We convert potential temperatures to thermal forcing (the difference between the in situ temperature and freezing point of seawater), using pressure and salinity to calculate the freezing point as

4 $${t}_{f}={a}_{0}S+{a}_{1}{S}^{3/2}+{a}_{2}{S}^{2}+{bp}$$

where ${t}_{f}$ is the freezing point temperature, $S$ is salinity, $p$ is pressure, and the remainder are constants (${a}_{0}$ = −0.0575, ${a}_{1}$ = 1.710523e^−^^3^, ${a}_{2}$ = −2.154996e^−^^4^, and $b$ = −7.52e^−^^4^)^76^. We calculated monthly anomalies in the thermal forcing over the continental shelf in front of Steenstrup (Fig. 2b). Here, we use sampling zones matching Wood et al.^12^ – specifically zone CE1 (Supplementary Fig. S5).

CMEMS data, as with all ocean reanalysis data, are subject to known limitations over shallow continental shelves^77^, lacking eddy resolution and not optimised to match the depth of the thermocline. As a result, we evaluate the CMEMS product by comparing the monthly average data to all available OMG CTDs that fall within zone CE1 between 2016 and 2020 (Supplementary Fig. S9). The mean difference between reanalysis and observational is −0.03 °C. This suggests that data has a moderate precision but relatively good accuracy at a spatially and temporally averaged scale. However, the in situ data is limited in temporal and spatial availability and is not sufficient to assess the biases of any seasonal or interannual trends over the study period.

### Bathymetry

Bathymetric data (Supplementary Figs. S4 and S5) is derived regionally from the International Bathymetric Chart of the Arctic Ocean (IBCAO) Grid v4.1^78^ and locally from multi-beam echo sounding (MBES) bathymetry data from the Oceans Melting Greenland (OMG) project^43^.

### Undercut modelling

The rate of undercutting (monthly average melt rate across the submerged calving face) is parameterised^10,12^ as

5 $${q}_{m}=\left(A\,h\,{q}_{{sg}}^{{\alpha }}\,B\right)T{F}^{\beta },$$

where $h$ is the average water depth across the calving front, set at 320 m based on OMG MBES data. ${TF}$ is the depth-averaged monthly ocean thermal forcing in the lower 60% of the water column, from CMEMS data. ${q}_{{sg}}$ is the basin-integrated monthly subglacial discharge averaged over the glacier front area (width 4000 m). The hydrological basin of Steenstrup is taken from Mankoff et al.^79^ with $k$ = 0.9, and the subglacial discharge represents the sum of (1) the basin-integrated monthly surface runoff is from RACMO2.3p2^41^ at 1 km resolution, statistically downscaled from 5.5 km; and (2) basin-integrated monthly subglacial melt, assumed constant, from Karlsson et al.^80^. Constants $A$ (3 × 10^4^), *α* (0.39), $B$ (0.15), and $\beta$ (1.18) are set following Rignot et al.^10^. The output, ${q}_{m}$, is the average across the submerged calving face, in metres per day. The mean nominal uncertainty is assigned at 26%^10,12^.

## Supplementary information

Supplementary Information

## Data Availability

Source data (terminus positions, ice discharge history, mélange data, custom ITS_LIVE annual velocity fields, ocean reanalysis data, and modelling inputs and results) necessary to replicate this study and the figures within have been deposited in a Zenodo repository and are openly available at 10.5281/zenodo.6903789^81^. ITS_LIVE scene-pair data are available from 10.5067/IMR9D3PEI28U^67^. ArcticDEM 2 m strips are available at 10.7910/DVN/OHHUKH^73^. The CMEMS Arctic Ocean Physics Reanalysis monthly product is available from 10.48670/moi-00007^42^. BedMachine v4 is available at 10.5067/VLJ5YXKCNGXO^74^. IBCAO v4 is available from https://www.gebco.net/^78^. OMG MBES gridded data is available from 10.5067/OMGEV-MBES1^43^. The monthly cumulative runoff product for Greenland from RACMO2.3p2 is freely available from the authors of ref. ^41^ upon request and without conditions.

## Code availability

Python code necessary to replicate the melt undercut modelling is included as a Jupyter Notebook alongside the data repository at 10.5281/zenodo.6903790^81^.
