# Supplementary material for: Atlantic water intrusion triggers rapid retreat and regime change at previously stable Greenland glacier
Source: Nat Commun. 2023 Apr 19;14:2151. doi: 10.1038/s41467-023-37764-7 (PMC10115864; doi:10.1038/s41467-023-37764-7)
Supplement: Supplementary file 1 — Supplementary Information [file 41467_2023_37764_MOESM1_ESM.pdf]

Supplement to ‘Atlantic Water intrusion triggers rapid retreat and regime change at previously stable Greenland glacier’

Chudley, T. R.[1]*, Howat, I. M.[1,2], King, M. D.[3], Negrete, A.[1]

[1] Byrd Polar and Climate Research Center, Ohio State University, Columbus, OH, USA
[2] School of Earth Sciences, Ohio State University, Columbus, OH, USA
[3] Polar Science Center, University of Washington, Seattle, WA, USA
* Now at Department of Geography, Durham University, Durham, UK

Correspondence: Tom Chudley (chudley.1@osu.edu)

Supplementary Text

Supplementary Figures 1 – 9

Supplementary Tables 1 – 2

Supplementary Text

Accuracy of bed data at Steenstrup

BedMachine v4 data reports that the front ~600 m of Steenstrup (based on 2016 terminus position) is ~20 m above sea level (fig. 3a), a value that is patently unphysical. BedMachine v4 data source values indicate that no mass conversation was applied in the sector, and the bed depth was inferred solely from krigging of input data. Hence, we suggest that the anomaly is likely due to the fact that OMG MBES data for Steenstrup, which is incorporated into BedMachine[1], includes the calving face within the bathymetry data[2] (fig. S3a), which is thus misinterpreted to become an erroneous sea-level anomaly in synthesised datasets. Manual inspection of the OMG MBES data suggests that the true depth of the calving front lies in the range ~300-360 m, based upon which we suggest the foremost ~1.8 km of the bed profile is unreliable (fig. 3a).

To further investigate the reliability of the gridded data, we examine Operation IceBridge Multichannel Coherent Radar Depth Sounder (MCoRDS) airborne flight lines[3], which were also incorporated into the final product (fig S7). These suggest that our estimation of the extent of unreliable BedMachine extent is accurate, but also that BedMachine data within ~13 km of the 2016 terminus is likely reasonable. This supports our assessment that a retrograde bedslope is likely a positive feedback influencing the rapid 2018-2021 retreat. However, with the bed between ~13 – ~22 km upstream being poorly resolved in the MCoRDS data, the future influence of the bed geometry on Steenstrup's retreat is uncertain.

Our finding that Steenstrup's front depth is underestimated in BedMachine v4 is consistent with Millan et al.[4], who integrate high-resolution OMG airborne gravimetry data to show that glaciers in SE GrIS have fjords hundreds of metres deeper than shown in BedMachine v3, and fewer shallow sills that limit access to AW. These erroneous data exist even though Steenstrup has a surprising amount of observations for a previously unremarkable glacier, including multiple IceBridge MCoRDS repeats and OMG MBES data. Many other glaciers suffer from a lack of observations entirely. Glaciers with notably long trunks bounded by valleys (typical for the south and central eastern sectors) are poorly represented by BedMachine due to a lack of observational data, with reported thicknesses no greater than tens of metres despite moving at a rates in excess of several metres per day. It is likely that the current literature, working largely from Greenland-wide synthesis datasets such as BedMachine, has underidentified vulnerable marine-terminating glaciers, with many other previously stable glaciers such as Steenstrup still potentially vulnerable.

Supplementary Figures

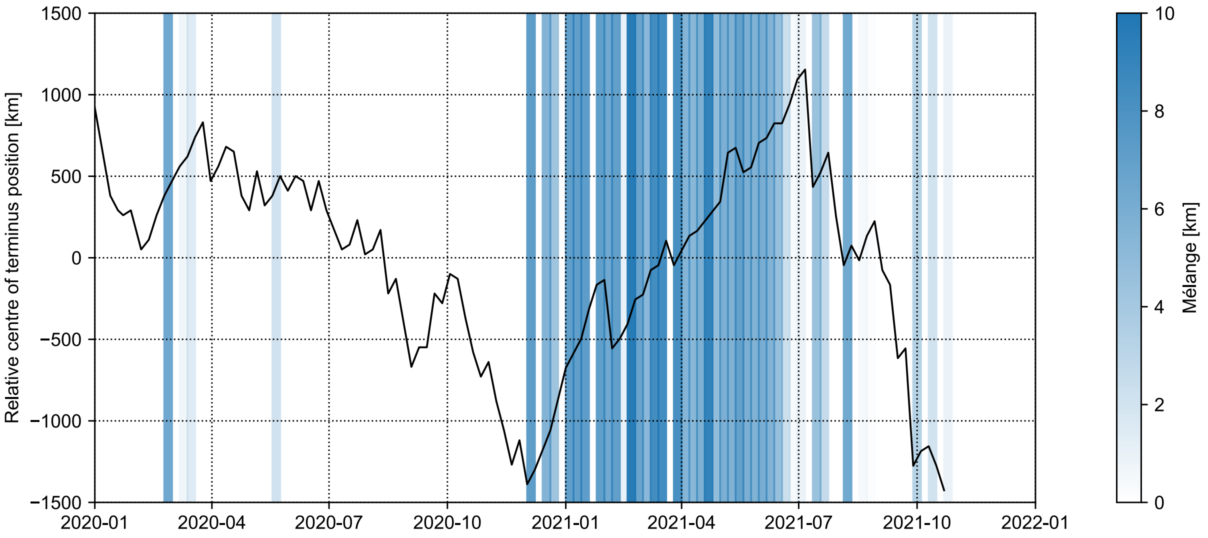

Figure S1: *Expanded version of fig. 2b showing terminus position (black line) and mélange presence and extent (blue shading) in 2020 and 2021.*

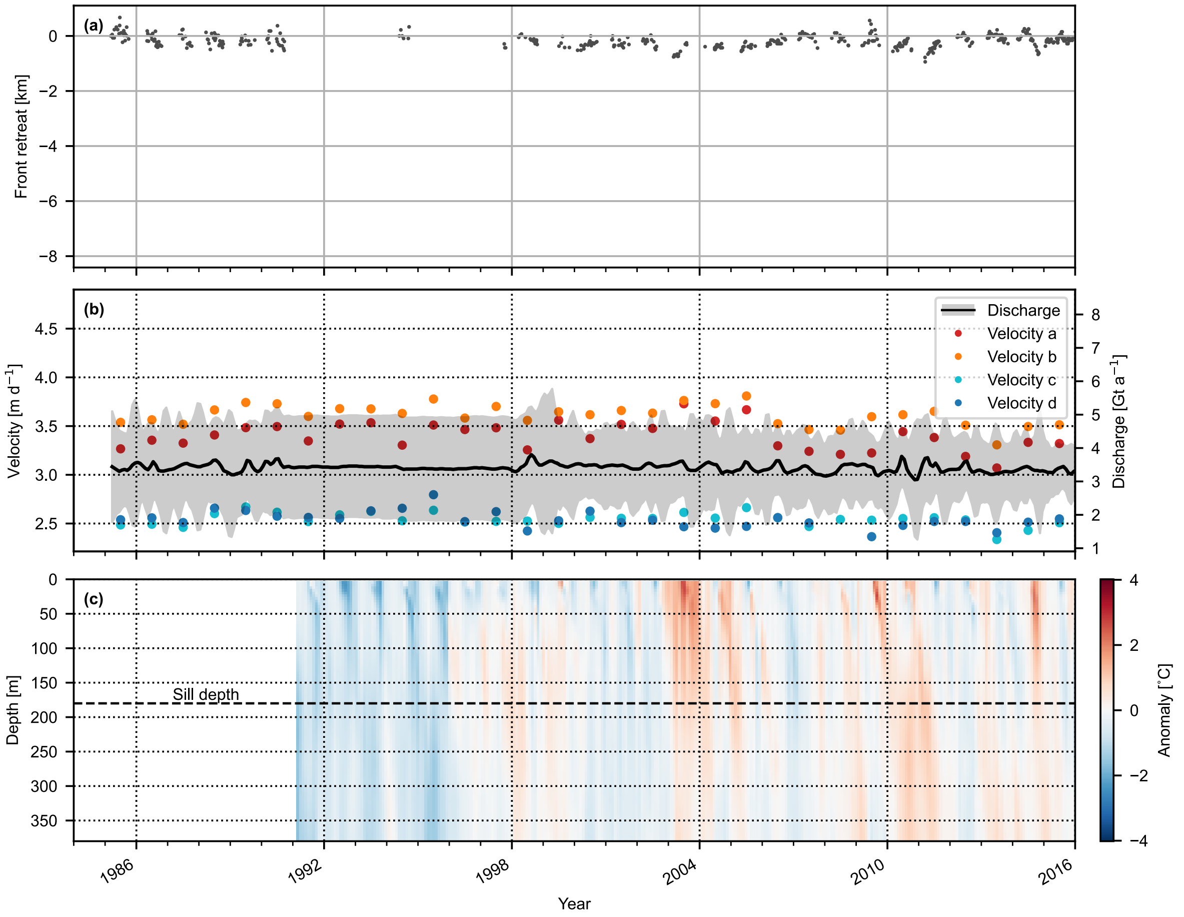

Figure S2: *Expanded version of figures 2a, 2c, and 2e, showing (a) front position; (b) discharge (with 2σ uncertainty) and annual mean velocity, and (c) CMEMS monthly mean ocean temperature anomaly between 1985 and 2016.*

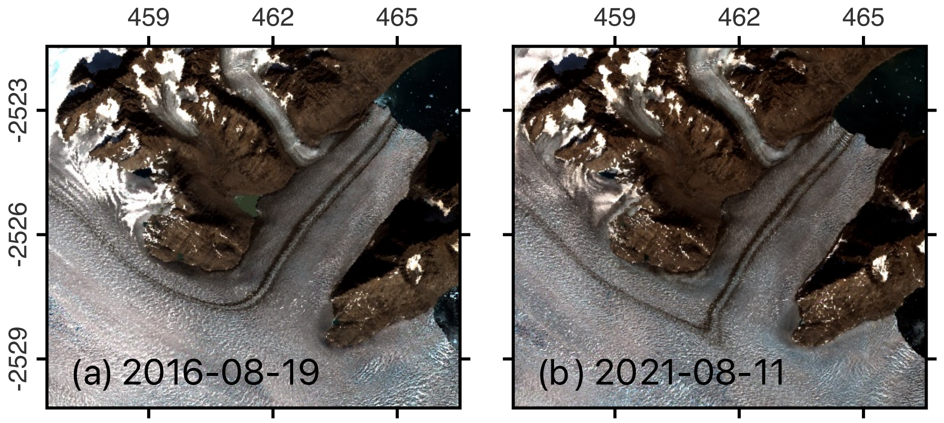

Figure S3: *Sentinel-2 imagery showing displacement of the medial moraine of the northern distributary of Steenstrup between 2016 and 2021 as flow is captured by the main trunk. Coordinates in units km of NSIDC Sea Ice Polar Stereographic North (EPSG:3413).*

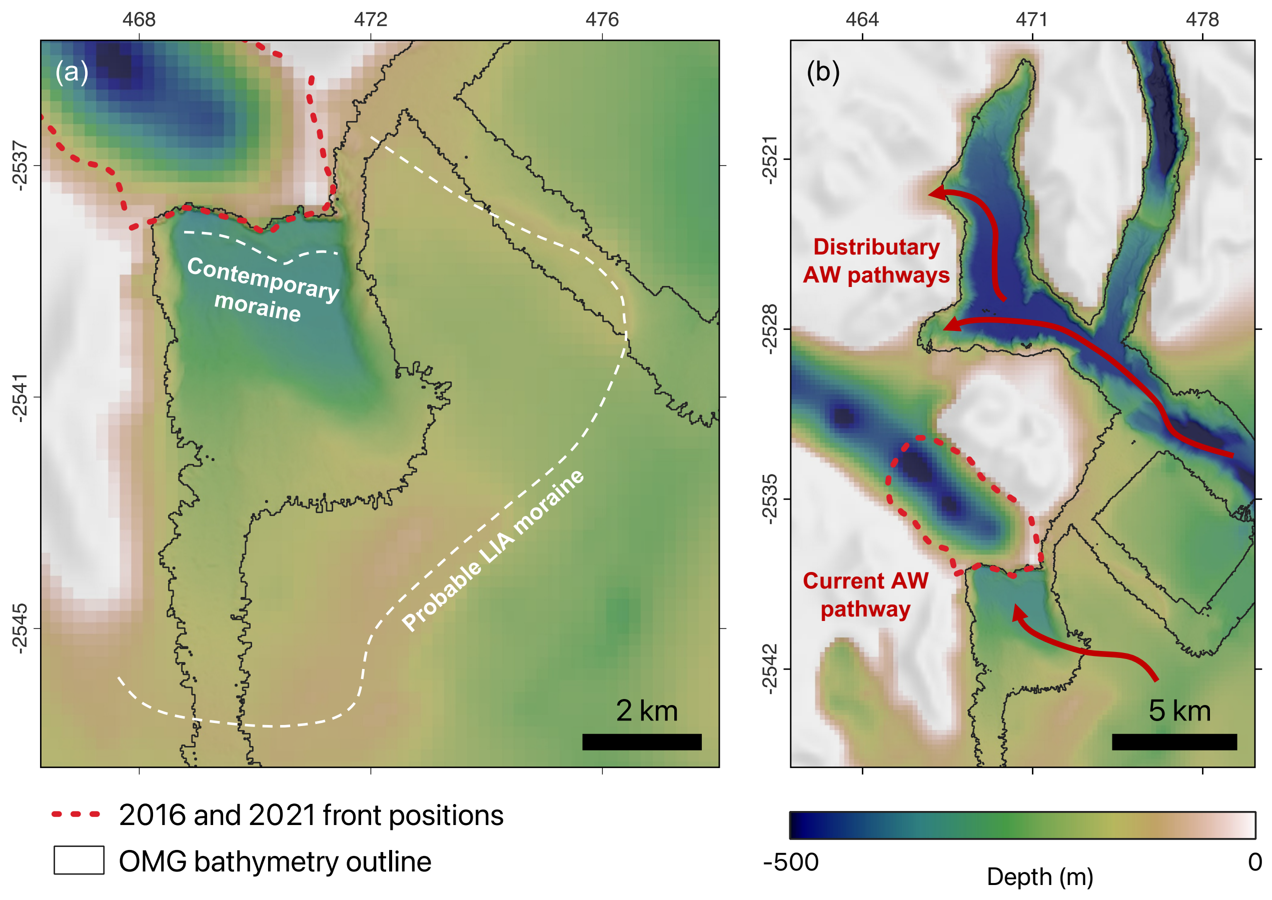

Figure S4: *(a) Hillshaded bathymetric data in front of Steenstrup from IBCAO v4 (which draws subglacial topography from BedMachine) and OMG MBES data. White dashed lines mark the contemporary and Little Ice Age (LIA) moraines identified by Batchelor* et al.[5]*. NB the artificial sea-level topography introduced into BedMachine at the 2016 terminus position due to OMG MBES incorporation of the calving front. (b) Potential alternative AW pathways through the distributaries. NB the inability of BedMachine to resolve ice depth at the northern distributary.*

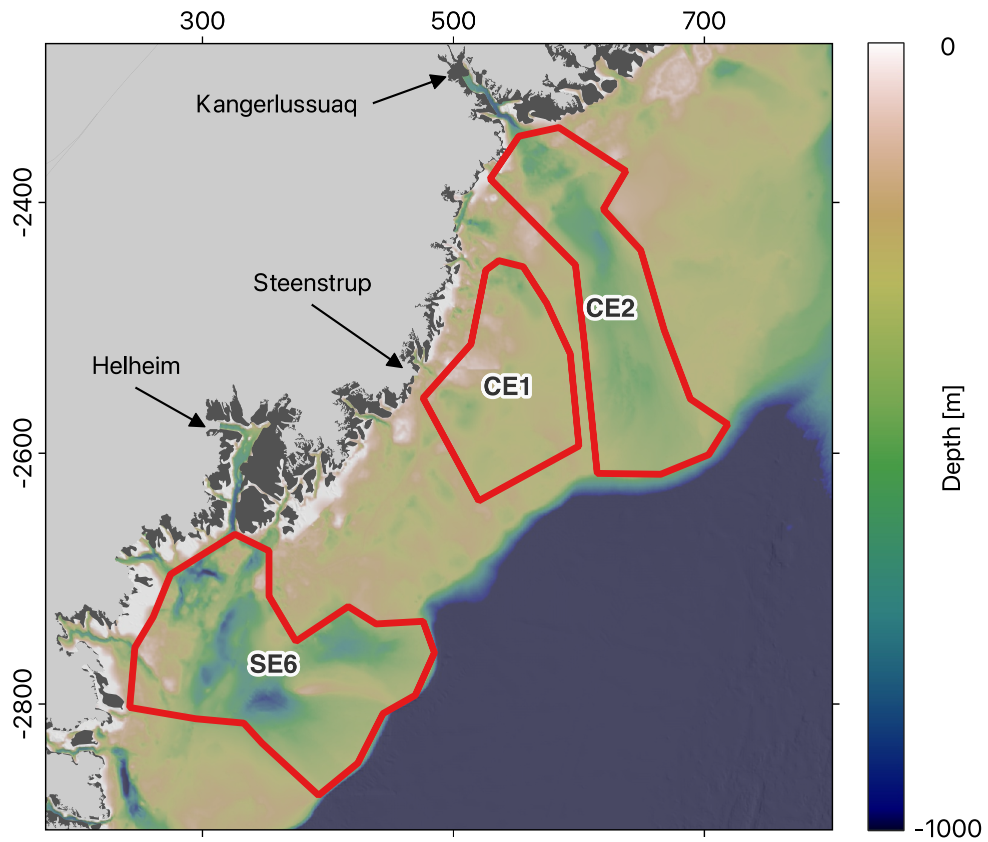

Figure S5: *Location of Wood* et al.[6] *sample zones. Background is hillshaded IBCAO bathymetry. Coordinates are in units km of NSIDC Sea Ice Polar Stereographic North (EPSG:3413).*

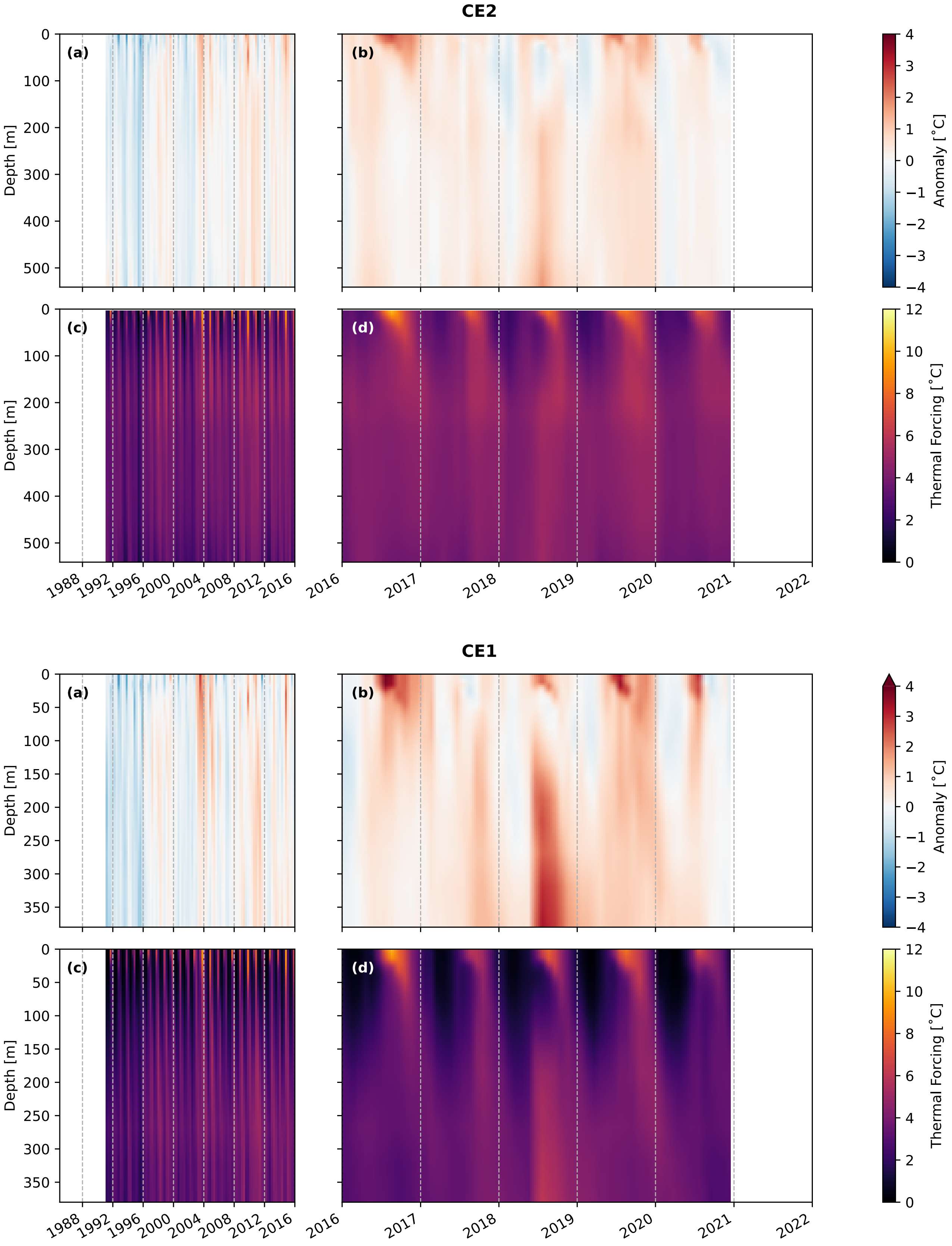

Figure S6: *Ocean temperature anomaly and thermal forcing for each of the CE2, CE1, and SE6 sample zones from Wood* et al.[6]*. For each subfigure: (a) Temperature anomaly from the 1992-2021 mean between 1992 and 2015. (b) Temperature anomaly from the 1992-2021 mean between 2016 and 2021. (c) Thermal forcing between 1992 and 2015. (d) Thermal forcing between 2016 and 2021.* ***(continues on next page)***

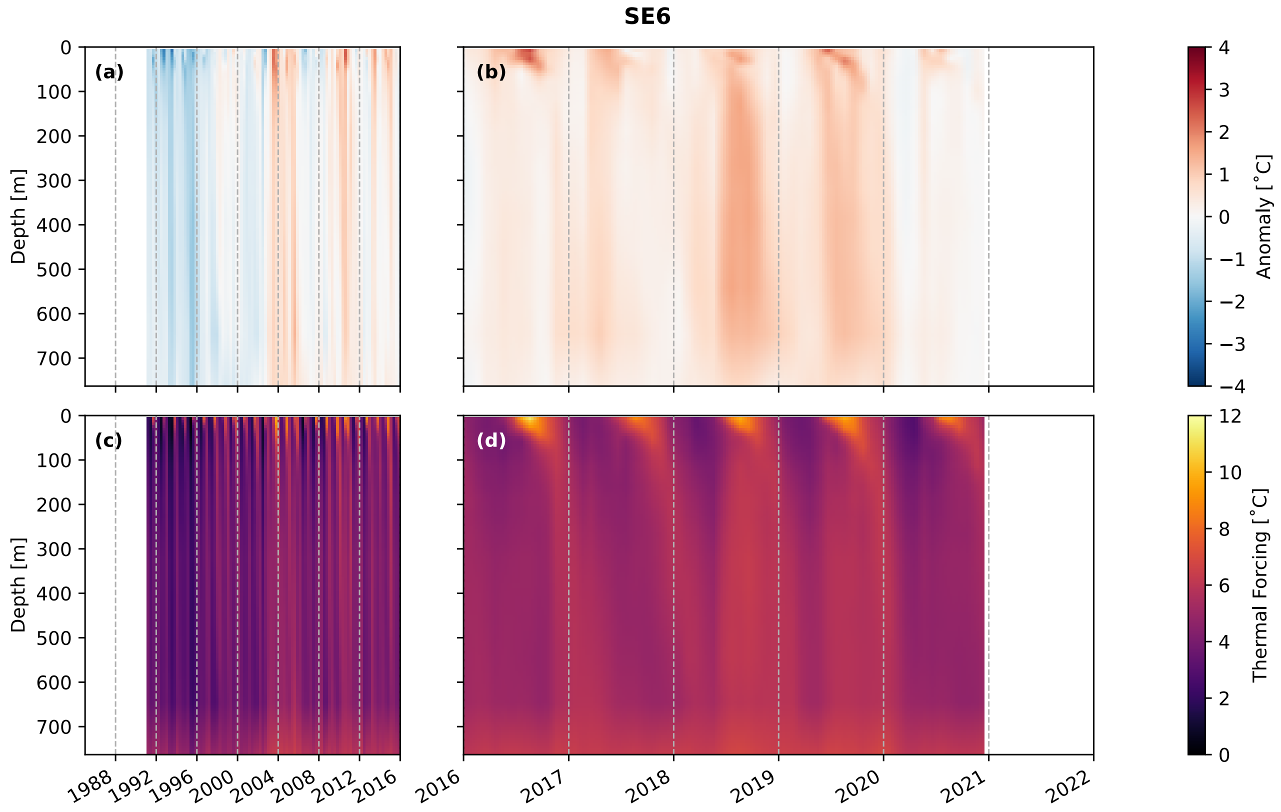

Figure S6: *(continued)*

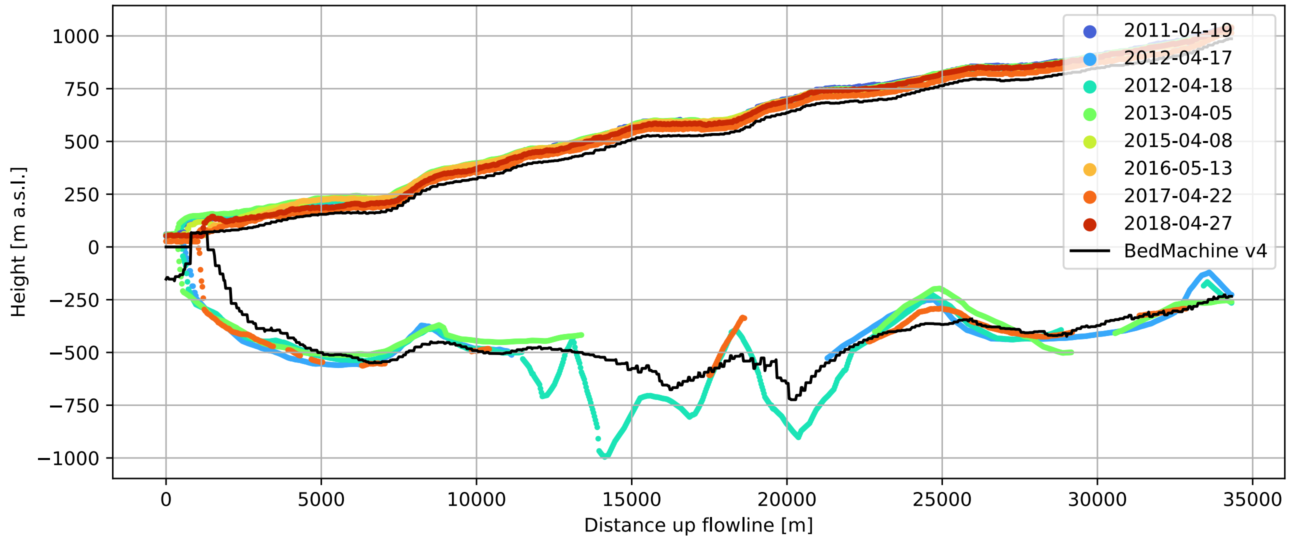

Figure S7: *Airborne MCoRDS data[3] visualising the surface and bed returns in comparison to BedMachine v4 data (black line).*

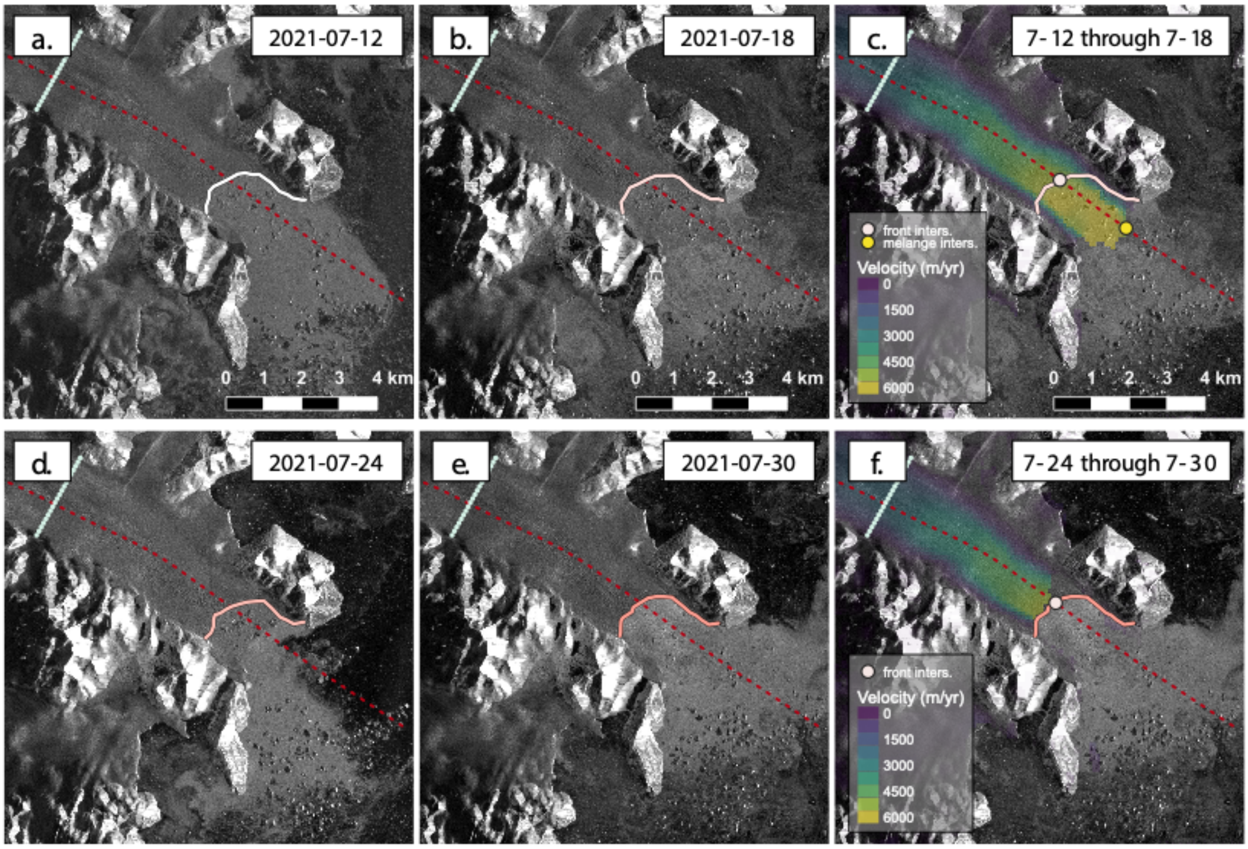

Figure S8: *Consecutive Sentinel-1 images for July 12th, 2021 (a) and July 18th, 2021 (b), and the corresponding velocity map between the two images in (c). The flux gate measurements coordinates are plotted upstream in light green, as well as an extended centre flowline as the dashed red line. The terminus position in each image is also delineated in white to pink shades with time, with circled markers indicating the intersection between both the terminus and outer extent of rigid melange with the centre flowline. Unlike the proglacial rigid melange shown in (c), velocity mapping between July 24 (d) and 30th (e) indicate the melange decorrelates between successive images and is nonrigid (f).*

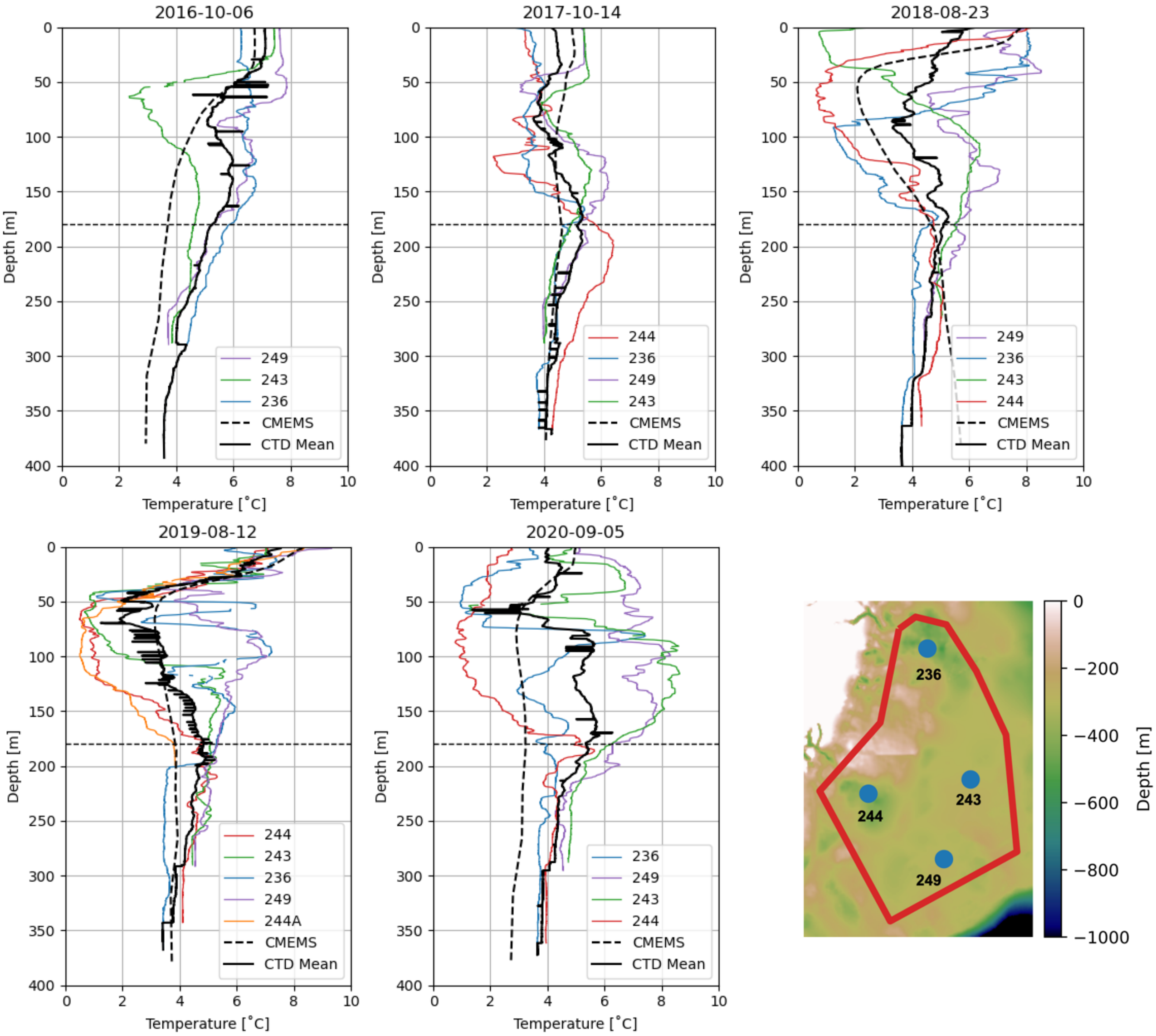

Figure S9: *OMG CTD drops, individual (coloured lines) and average (solid black line) from drop locations, that fall within the CE1 sample zone (fig S5), compared to the CMEMS monthly average reanalysis product across the sample zone (dotted black line). Horizontal dashed black line refers to the absolute lower limit of Steenstrup's proglacial sill. CTD labels refer to the drop location number: 243, 236, 244, and 249, which are visualised within the CE1 sample zone in the inset map.*

Supplementary Tables

Table S1: *Positions of sampling points presented in Fig 1a and Fig 2b, in coordinates of NSIDC Sea Ice Polar Stereographic North (EPSG:3413).*

Point	X	Y
a	464895	-2533001
b	460732	-2530257
c	456475	-2527582
d	451777	-2526768

Table S2: *Date and IDs of ArcticDEM strips used*

Date	ArcticDEM 2m Strip
2016-03-27	WV01_20160327_102001004BC7AC00_102001004AC12E00_2m_lsf
2016-04-04	WV01_20160404_102001004AA8F800_102001004D67D900_2m_lsf
2016-08-03	WV01_20160803_1020010054389200_10200100537DE100_2m_lsf
2017-06-07	WV01_20170607_1020010063D4CE00_10200100617E7F00_2m_lsf
2017-08-05	WV01_20170805_1020010065C0F200_102001006260ED00_2m_lsf
2018-04-05	WV01_20180405_1020010072328700_102001007149E600_2m_lsf
2018-04-23	WV03_20180423_104001003A7FE400_104001003A9F3100_2m_lsf
2018-05-30	WV03_20180530_104001003DC69400_104001003E486E00_2m_lsf
2019-05-11	WV03_20190511_104001004BBA4500_104001004C5DCC00_2m_lsf
2019-06-14	WV01_20190614_102001008B302C00_1020010087237C00_2m_lsf
2019-07-22	WV01_20190722_1020010086AC6800_1020010085418C00_2m_lsf
2020-07-09	WV01_20200709_102001009A689B00_102001009B63B200_2m_lsf
2020-07-14	WV01_20200714_102001009BEB8300_102001009CB09500_2m_lsf
2021-07-31	WV02_20210731_10300100C359CF00_10300100C37C8000_2m_lsf
2021-08-14	WV02_20210814_10300100C3611400_10300100C4831700_2m_lsf

Supplementary References

1. Morlighem, M. *et al.* BedMachine v3: Complete Bed Topography and Ocean Bathymetry Mapping of Greenland From Multibeam Echo Sounding Combined With Mass Conservation. *Geophys. Res. Lett.* 2017GL074954 (2017) doi:10.1002/2017GL074954.
2. Oceans Melting Greenland. Oceans Melting Greenland Bathymetric Survey - South East Greenland 2016 Operations Report: Appendix A - Daily Field Reports. (2016).
3. Paden, J., Leuschen, Carl, Rodriguez-Morales, F. & Hale, R. IceBridge MCoRDS L2 Ice Thickness, Version 1. (2010) doi:10.5067/GDQ0CUCVTE2Q.
4. Millan, R. *et al.* Vulnerability of Southeast Greenland Glaciers to Warm Atlantic Water From Operation IceBridge and Ocean Melting Greenland Data. *Geophys. Res. Lett.* **45**, 2688–2696 (2018).
5. Batchelor, C. L., Dowdeswell, J. A., Rignot, E. & Millan, R. Submarine Moraines in Southeast Greenland Fjords Reveal Contrasting Outlet-Glacier Behavior since the Last Glacial Maximum. *Geophys. Res. Lett.* **46**, 3279–3286 (2019).
6. Wood, M. *et al.* Ocean forcing drives glacier retreat in Greenland. *Sci. Adv.* **7**, eaba7282 (2021).
